# Randomized Controlled Trial of the Electrocardiographic Effects of Four Antimalarials for Pregnant Women with Uncomplicated Malaria on the Thailand-Myanmar Border

**DOI:** 10.1128/AAC.02473-20

**Published:** 2021-03-18

**Authors:** Makoto Saito, Widi Yotyingaphiram, Zillah Cargill, Mary Ellen Gilder, Aung Myat Min, Aung Pyae Phyo, Thi Dar San, Hilda Poe, Cindy Chu, Nicholas J. White, François Nosten, Rose McGready

**Affiliations:** a Shoklo Malaria Research Unit, Mahidol-Oxford Tropical Medicine Research Unit, Faculty of Tropical Medicine, Mahidol University, Mae Sot, Thailand; b Centre for Tropical Medicine and Global Health, Nuffield Department of Medicine, University of Oxford, Oxford, United Kingdom; c Division of Infectious Diseases, Advanced Clinical Research Center, Institute of Medical Science, University of Tokyo, Tokyo, Japan; d Maidstone Hospital, Maidstone and Tunbridge Wells NHS Trust, Kent, United Kingdom; e Department of Family Medicine, Chiang Mai University, Chiang Mai, Thailand; f Mahidol-Oxford Tropical Medicine Research Unit (MORU), Faculty of Tropical Medicine, Mahidol University, Bangkok, Thailand

**Keywords:** QT prolongation, cardiotoxicity, chloroquine, JT interval, lumefantrine, malaria, mefloquine, piperaquine, pregnancy, safety

## Abstract

Quinoline antimalarials cause drug-induced electrocardiographic QT prolongation, a potential risk factor for torsade de pointes. The effects of currently used antimalarials on the electrocardiogram (ECG) were assessed in pregnant women with malaria. Pregnant women with microscopy-confirmed parasitemia of any malaria species were enrolled in an open-label randomized controlled trial on the Thailand-Myanmar border from 2010 to 2016. Patients were randomized to the standard regimen of dihydroartemisinin-piperaquine (DP) or artesunate-mefloquine (ASMQ) or an extended regimen of artemether-lumefantrine (AL+). Recurrent Plasmodium vivax infections were treated with chloroquine. Standard 12-lead electrocardiograms were assessed on day 0, 4 to 6 h following the last dose, and day 7. QT was corrected for the heart rate by a linear mixed-effects model-derived population-based correction formula (QTcP = QT/RR^0.381^). A total of 86 AL+, 82 ASMQ, 88 DP, and 21 chloroquine-treated episodes were included. No patients had an uncorrected QT interval nor QTcP of >480 ms at any time. QTcP corresponding to peak drug concentration was longer in the DP group (adjusted predicted mean difference, 17.84 ms; 95% confidence interval [CI], 11.58 to 24.10; *P* < 0.001) and chloroquine group (18.31 ms; 95% CI, 8.78 to 27.84; *P* < 0.001) than in the AL+ group, but not different in the ASMQ group (2.45 ms; 95% CI, −4.20 to 9.10; *P* = 0.47) by the multivariable linear mixed-effects model. There was no difference between DP and chloroquine (*P* = 0.91). QTc prolongation resulted mainly from widening of the JT interval. In pregnant women, none of the antimalarial drug treatments exceeded conventional thresholds for an increased risk of torsade de pointes.

## Abstract (precis)

Quinoline antimalarials cause drug-induced electrocardiograph QT prolongation, a potential risk factor for torsade de pointes. The effects of currently used antimalarials on the electrocardiogram (ECG) were assessed in pregnant women with malaria.

## INTRODUCTION

Quinoline and structurally related antimalarials have pharmacological activity on the cardiovascular system. Quinidine, the dextrorotatory diastereomer of quinine, has been used mainly as a class 1a antiarrhythmic rather than as an antimalarial, despite its greater antimalarial activity than quinine (1, 2). Halofantrine was withdrawn because, after registration, it was found to cause marked prolongation of QT interval in therapeutic doses (3), and it was associated with sudden cardiac deaths (1, 3, 4). Although drug-induced QT prolongation has been reported for other antimalarials, namely, chloroquine, amodiaquine, mefloquine, lumefantrine, and piperaquine (1, 5–8), previous studies conducted in malaria patients and also in healthy adult volunteers have found that the cardiovascular safety profile of currently used antimalarials is generally reassuring.

Pregnant women are a subgroup for whom drug toxicity may differ from other individuals because of the physiological and pharmacokinetic/pharmacodynamic differences resulting from pregnancy itself (9–11). The QT-prolonging effect of medications is consistently higher in women than in men (1, 7, 12), while changes in sex hormones during pregnancy can shorten the QT interval (13). There have been few detailed electrocardiographic studies of antimalarials in pregnancy. Furthermore, more than half of the pregnant women studied in reports of electrocardiographic effects were healthy volunteers taking antimalarials as prevention, rather than women with malaria parasitemia taking treatment (14–27).

Malaria infection itself affects the QT interval, and defervescence contributes to a substantial reduction in heart rate and lengthening of the QT interval between the acute admission and 3 days later when drug levels are at their highest (1). Therefore, in patients with malaria parasitemia, differences in QT intervals before and after drug administration are not attributable only to the effect of drugs. Comparing the QT interval changes between malaria patients assigned to different treatment regimens allows assessment of the impact of different drugs without confounding from the resolving malaria infection itself. QT prolongation can be caused by delaying ventricular depolarization (class 1c effect) or repolarization (class 3 effect) (1). Differentiation, by dividing QT into the QRS and JT intervals, is important, as class 3 antiarrhythmics can cause torsades de pointes, and the risk has been reported to be higher in women (28). Few studies on antimalarials have differentiated these two effects.

We compared QT and JT intervals before and after four different antimalarials (extended regimen of artemether-lumefantrine [AL+], artesunate-mefloquine [ASMQ], dihydroartemisinin-piperaquine [DP], and chloroquine [CHQ]) in pregnant women who were enrolled in a randomized controlled trial (RCT) of antimalarial treatments for uncomplicated malaria in pregnancy on the Thailand-Myanmar border.

## RESULTS

### Baseline characteristics.

Among 511 pregnant women enrolled in the RCT, 256 pregnant women (86 AL+, 82 ASMQ, and 88 DP) had electrocardiogram (ECG) assessments (Fig. S1 in the supplemental material). During the follow-up, there were 71 recurrences of Plasmodium vivax before delivery that were treated with CHQ, and 21 of them were assessed by ECG. Twenty patients had two episodes where ECGs were assessed; the median interval between two episodes was 67 (range, 27 to 133) days.

From 279 malaria episodes in 259 pregnant women, 754 ECG records were performed: at baseline before drug administration (day 0; *n* = 273), at 4 to 6 h following the last dose (defined as day peak; *n* = 256), and on day 7 or after (day 7; *n* = 225). The recording speed of the ECG was 50 mm/s in 98.7% (744/754) of the records. Some records were excluded from the analyses, including ECG assessed after drug administration on day 0 (*n* = 2); <6 leads assessed on day 0 (*n* = 2), on day peak (*n* = 2), or on day 7 (*n* = 3); and poor quality on day 0 (*n* = 1). Appropriate ECGs on all 3 days were available in 205 episodes.

The mean age of the pregnant women with uncomplicated malaria was 25.6 years (standard deviation [SD], 6.9; range, 18 to 45), and the mean gravidity was 2.9 (SD, 2.0; maximum, 10). The mean estimated gestational age (EGA) on day 0 was 26.0 weeks (SD, 8.3; range, 7.0 to 40.1). There were 79 Plasmodium falciparum monoinfections, 194 P. vivax monoinfections, 5 coinfections of P. falciparum and P. vivax, and 1 Plasmodium malariae monoinfection. Only 30.2% (84/278) were febrile (body temperature >37.5°C) at presentation.

There were no apparent differences in baseline characteristics among the three randomized treatment groups (Table 1). CHQ was used exclusively for mildly symptomatic recurrences of P. vivax monoinfection. There were no apparent differences between those who were assessed by ECG and those who were not (Table S1).

**TABLE 1:** Baseline characteristics on day 0 of pregnant women who were assessed by ECG

Characteristic	Data for treatment group:									
	All participants		AL+^*a*^		ASMQ		DP		CHQ	
	No. of women	Mean (SD) or % (no. of women)	No. of women	Mean (SD) or % (no. of women)	No. of women	Mean (SD) or % (no. of women)	No. of patients	Mean (SD) or % (no. of patients)	No. of patients	Mean (SD) or % (no. of patients)
Age (yrs)	259^*b*^	25.6 (6.9)	86	25.1 (7.1)	82	25.8 (6.6)	88	25.5 (6.9)	21	25.7 (7.6)
Gravidity	259^*b*^	2.9 (2.0)	86	2.9 (2.2)	82	3.0 (2.1)	88	2.7 (1.8)	21	3.1 (2.0)
Height (cm)	259^*b*^	151.0 (5.6)	86	151.0 (6.1)	82	151.4 (4.9)	88	150.9 (5.9)	21	150.8 (5.2)
Smoking	259^*b*^	20.8% (54)	86	23.3% (20)	82	18.3% (15)	88	19.3% (17)	21	28.6% (6)
Gestational age (wks)	279	26.0 (8.3)	86	25.7 (8.6)	82	26.5 (8.9)	88	24.6 (7.8)	21	29.8 (5.3)
Weight (kg)	276	52.3 (8.0)	86	51.2 (7.8)	82	53.1 (7.6)	88	51.8 (8.3)	20	55.5 (8.0)
Fever (>37.5°C)	278	30.2% (84)	86	33.7% (29)	82	34.1% (28)	88	27.3% (24)	21	14.3% (3)
Heart rate (per min)	273	93.6 (16.2)	84	96.2 (17.2)	80	95.6 (16.0)	88	91.0 (15.7)	19	86.8 (12.6)
Hematocrit (%)	279	32.4 (3.7)	86	32.3 (3.3)	82	32.8 (4.3)	88	32.3 (3.8)	21	31.9 (2.7)
Malaria species infection type	279		86		82		88		21	
P. falciparum monoinfection		28.3% (79)		30.2% (26)		35.4% (29)		25.0% (22)		0.0% (0)
P. vivax monoinfection		69.5% (194)		68.6% (59)		62.2% (51)		71.6% (63)		100.0% (21)
P. falciparum plus P. vivax		1.8% (5)		1.2% (1)		2.4% (2)		2.3% (2)		0.0% (0)
*Plasmodium malariae* monoinfection		0.4% (1)		0.0% (0)		0.0% (0)		1.1% (1)		0.0% (0)
Asexual parasite load/μl	279	10,262.9 (21,418.2)	86	10,304.4 (19,886.1)	82	13,090.7 (25,924.4)	88	9,621.2 (20,502.7)	21	2,400.8 (6,403.9)
Presence of gametocyte	279	48.4% (135)	86	47.7% (41)	82	40.2% (33)	88	52.3% (46)	21	66.7% (14)
Anorexia	279	38.0% (106)	86	38.4% (33)	82	40.2% (33)	88	37.5% (33)	21	23.8% (5)
Nausea	279	31.5% (88)	86	36.0% (31)	82	37.8% (31)	88	29.5% (26)	21	0.0% (0)
Vomiting	279	16.1% (45)	86	12.8% (11)	82	24.4% (20)	88	14.8% (13)	21	0.0% (0)
Dizziness	279	63.1% (176)	86	64.0% (55)	82	61.0% (50)	88	68.2% (60)	21	47.6% (10)
Diarrhea	279	2.2% (6)	86	3.5% (3)	82	0.0% (0)	88	3.4% (3)	21	0.0% (0)
Palpitation	279	31.5% (88)	86	33.7% (29)	82	30.5% (25)	88	37.5% (33)	21	0.0% (0)
Fatigue	279	53.8% (150)	86	50.0% (43)	82	56.1% (46)	88	55.7% (49)	21	47.6% (10)

a AL+, extended artemether-lumefantrine; ASMQ, artesunate-mefloquine; CHQ, chloroquine; DP, dihydroartemisinin-piperaquine.

b Only the first episode was included.

### Heart rate, QTu, QTcF, and QTcB.

The mean heart rate on day 0 was 93.4 beats per minute (SD, 16.2), 78.6 (SD, 11.8) on day peak, and 86.2 (SD, 12.9) on day 7. Bradycardia (heart rate < 60 beats/min) on day peak was observed in 13 patients, including 9.8% (8/82) of AL+, 2.6% (2/78) of ASMQ, 2.5% (2/79) of DP, and 5.9% (1/17) of CHQ. Of the symptoms that might be associated with bradycardia, only 2 of the 13 patients complained of dizziness (one each in DP and ASMQ). None of them had vomited. There were no maternal deaths nor cases with life-threatening arrhythmias. On day 0, the mean uncorrected QT (QTu) was 334.3 ms (SD, 27.4), QT corrected by the Fridericia method (QTcF) was 384.8 ms (SD, 20.4), and by the Bazett method (QTcB) was 413.4 ms (SD 23.4) (Table S2). There were no apparent differences on day 0 among the treatment groups. No patients had QTu or QTcF longer than 480 ms at any time, but QTcB was longer than 480 ms in three women, with a maximum value of 493 ms.

Compared with the baseline, a QTcF increase of >30 ms was observed on day peak in 10.1% (8/79) of AL+, 16.4% (12/73) of ASMQ, 37.5% (6/16) of CHQ, and 40.4% (31/77) of DP (Table 2). A QTcF increase of >60 ms was observed only in DP (5.2%, 4/77), with a maximum of 68 ms. Compared with the baseline, a QTcB increase of >30 ms was observed on day peak in 7.6% (6/79) of AL+, 5.5% (4/73) of ASMQ, 25.0% (4/16) of CHQ, and 30.2% (24/77) of DP recipients (Table 2). A QTcB increase of >60 ms was observed in DP (2.6%, 2/77) and CHQ (6.3%, 1/16), with a maximum of 72 ms.

**TABLE 2:** Difference in QT intervals by various correction methods from the baseline

Measurement (day peak − day 0)	Data for treatment group:									
	All		AL+^*a*^		ASMQ		DP		CHQ	
	Total	Mean (SD) or % (no. of women)	Total	Mean (SD) or % (no. of women)	Total	Mean (SD) or % (no. of women)	Total	Mean (SD) or % (no. of patients)	Total	Mean (SD) or % (no. of patients)
ΔQT uncorrected (ms)	245	34.5 (26.4)	79	32.0 (27.4)	73	29.6 (27.0)	77	41.4 (23.2)	16	36.3 (23.9)
QT >30 ms	245	64.5% (158)	79	62.0% (49)	73	53.4% (39)	77	76.6% (59)	16	68.8% (11)
QT >60 ms	245	19.6% (48)	79	12.7% (10)	73	21.9% (16)	77	24.7% (19)	16	18.8% (3)
Δ QTc Fridericia (ms)	245	15.84 (20.9)	79	9.76 (18.9)	73	9.02 (19.9)	77	26.6 (20.0)	16	25.3 (14.5)
QTc Fridericia >30 ms	245	23.3% (57)	79	10.1% (8)	73	16.4% (12)	77	40.3% (31)	16	37.5% (6)
QTc Fridericia >60 ms	245	1.6% (4)	79	0.0% (0)	73	0.0% (0)	77	5.2% (4)	16	0.0% (0)
Δ QTc Bazett (ms)	245	4.58 (24.96)	79	−3.54 (23.4)	73	−3.45 (23.0)	77	17.6 (22.9)	16	18.7 (18.7)
QTc Bazett >30 ms	245	15.5% (38)	79	7.6% (6)	73	5.5% (4)	77	31.2% (24)	16	25.0% (4)
QTc Bazett >60 ms	245	1.2% (3)	79	0.0% (0)	73	0.0% (0)	77	2.6% (2)	16	6.3% (1)
Δ QTcP (ms)	245	12.7 (21.5)	79	6.11 (19.5)	73	5.60 (20.2)	77	24.1 (20.4)	16	23.5 (15.0)
QTcP >30 ms	245	19.6% (48)	79	10.1% (8)	73	8.2% (6)	77	36.4% (28)	16	37.5% (6)
QTcP >60 ms	245	1.6% (4)	79	0% (0)	73	0% (0)	77	5.2% (4)	16	0% (0)

a AL+, extended artemether-lumefantrine; ASMQ, artesunate-mefloquine; CHQ, chloroquine; Δ, difference; DP, dihydroartemisinin-piperaquine; QTc Fridericia, QT interval corrected by Fridericia method; QTc Bazett, QT interval corrected by Bazett method; QTcP, QT interval corrected by population-based correction (QT/RR^0.381^).

### QTc by population-based correction.

Using the day 0 data, the population-based heart rate correction formula obtained was QTcP = QTu/RR^0.381^. QTc by population-based correction (QTcP) was no longer associated with heart rate by linear regression analysis (coefficient, −0.016; 95% confidence interval [CI], −0.17 to 0.14; *P* = 0.84), while QTcF (coefficient, −0.20; 95% CI, −0.35 to −0.06; *P* = 0.007) and QTcB (coefficient, 0.49; 95% CI, 0.33 to 0.65; *P* < 0.001) were associated with heart rate on day 0 (Fig. S2). No association between QTcP and heart rate was also observed for day peak and day 7; thus, QTcP was used for further analyses.

### Factors associated with QTcP.

In the univariable analysis, QTcP on day peak was longer in DP (mean difference, 17.49 ms; 95% CI, 12.37 to 22.62; *P* < 0.001) and CHQ (13.62 ms; 95% CI, 5.41 to 21.82; *P* = 0.001) than AL+, but no different in ASMQ (−0.37 ms; 95% CI, −5.54 to 4.81; *P* = 0.89). On day 7, there was no difference among the treatment groups (*P* = 0.40).

In the multivariable analysis (Table 3), QTcP on day peak was longer in DP (adjusted mean difference, 17.84 ms; 95% CI, 11.58 to 24.10; *P* < 0.001) and CHQ (18.31 ms; 95% CI, 8.78 to 27.84; *P* < 0.001) than AL+, but not different in ASMQ (2.45 ms; 95% CI, −4.20 to 9.10; *P* = 0.47). There was no difference between DP and CHQ (*P* = 0.91). On day peak, QTcP was longer than day 0 in all treatment groups; the adjusted mean difference from day 0 was 6.78 ms (95% CI, 1.36 to 12.19) in AL+, 9.23 ms (95% CI, 3.86 to 14.59) in ASMQ, 24.62 ms (95% CI, 20.13 to 29.10) in DP, and 25.09 ms (95% CI, 16.66 to 33.52) in CHQ.

**TABLE 3:** Factors associated with QTcP by multilevel univariable and multivariable linear regression^*a*^

Variable	Univariable linear regression results			Multivariable linear regression results		
	No. of patients	Mean difference (95% CI)	*P* value	No. of patients	Mean difference (95% CI)	*P* value
Time and treatment type (interaction)						
Day peak						
AL+	70	Reference		70	Reference	
ASMQ	74	−0.37 (−5.54 to 4.81)	0.89	76	2.45 (−4.20 to 9.10)	0.47
DP	79	17.49 (12.37 to 22.62)	<0.001	79	17.84 (11.58 to 24.10)	<0.001
CHQ	15	13.62 (5.41 to 21.82)	0.001	14	18.31 (8.78 to 27.84)	<0.001
Day 7						
AL+	64	Reference		64	Reference	
ASMQ	66	−0.05 (−5.54 to 5.44)	0.99	67	−0.05 (−5.59 to 5.48)	0.99
DP	62	−4.06 (−9.53 to 1.42)	0.15	62	−5.11 (−10.70 to −0.48)	0.07
CHQ	20	−0.13 (−7.72 to 7.45)	0.97	20	1.07 (−6.64 to 8.77)	0.79
Day (for AL+)						
0	83	Reference		83	Reference	
Peak	64	6.28 (2.49 to 10.06)	0.001	64	6.78 (1.36 to 12.19)	0.01
7	70	3.57 (−0.44 to 7.57)	0.081	70	5.64 (0.76 to 10.52)	0.02
Age	748	0.53 (0.25 to 0.81)	<0.001	720	0.53 (0.24 to 0.81)	<0.001
Gravidity	748	1.76 (0.80 to 2.73)	<0.001			
Height	748	0.06 (−0.29 to 0.42)	0.72			
Smoking	154/748	0.78 (−4.21 to 5.77)	0.76			
EGA	748	−0.43 (−0.65 to −0.21)	<0.001	720	−0.40 (−0.62 to −0.18)	<0.001
Weight	745	0.004 (−0.25 to 0.26)	0.97			
Fever (>37.5°C)	80/683	2.57 (−1.68 to 6.82)	0.24			
Body temperature	679	−1.47 (−4.07 to 1.13)	0.27			
Heart rate	748	0.07 (−0.03 to 0.16)	0.18			
Hematocrit	584	−0.33 (−0.78 to 0.12)	0.15			
Malaria species infection						
Negative	438	Reference				
P. falciparum monoinfection	110	0.09 (−5.58 to 5.77)	0.97			
P. vivax monoinfection	192	−1.35 (−7.35 to 4.66)	0.66			
P. falciparum plus P. vivax	5	−3.71 (−18.42 to 11.00)	0.62			
*P. malariae* monoinfection	1	0.47 (−30.77 to 31.70)	0.98			
Asexual parasitemia	746	0.12 (0.03 to 0.21)	0.007	720	0.11 (0.02 to 0.19)	0.02
Gametocytemia	135/747			135/720		
Between women		16.63 (6.17 to 27.10)	0.002		13.53 (3.17 to 23.90)	0.01
Within the same woman		−0.79 (−4.77 to 3.19)	0.70		−1.08 (−5.06 to 2.91)	0.60
Anorexia	135/721					
Between women		8.80 (1.69 to 15.92)	0.02			
Within the same woman		−2.10 (−6.27 to 2.08)	0.33			
Vomiting	55/723	5.23 (0.41 to 10.06)	0.03			
Diarrhea	5/722	27.49 (12.25 to 42.73)	<0.001	5/720	23.16 (8.18 to 38.14)	0.002
Time ECG assessed						
0:00 to 5:59	39	2.31 (−4.28 to 8.91)	0.49	36	3.13 (−3.68 to 9.93)	0.37
6:00 to 11:59	313	−1.69 (−4.78 to 1.40)	0.29	296	−0.32 (−3.47 to 2.83)	0.84
12:00 to 17:59	309	Reference		302	Reference	
18:00 to 23:59	87	−2.07 (−6.74 to 2.60)	0.39	86	−2.85 (−7.49 to 1.79)	0.23

a AL+, extended artemether-lumefantrine; ASMQ, artesunate-mefloquine; CHQ, chloroquine; CI, confidence interval; DP, dihydroartemisinin-piperaquine; ECG, electrocardiogram; EGA, estimated gestational age. *P* value by Wald test. Random-slope models were used for accounting for within-person correlation. Day and interaction between day and treatment were included in the models as forced variables. Multivariable model includes all the variables listed in the column.

**FIG 1:**
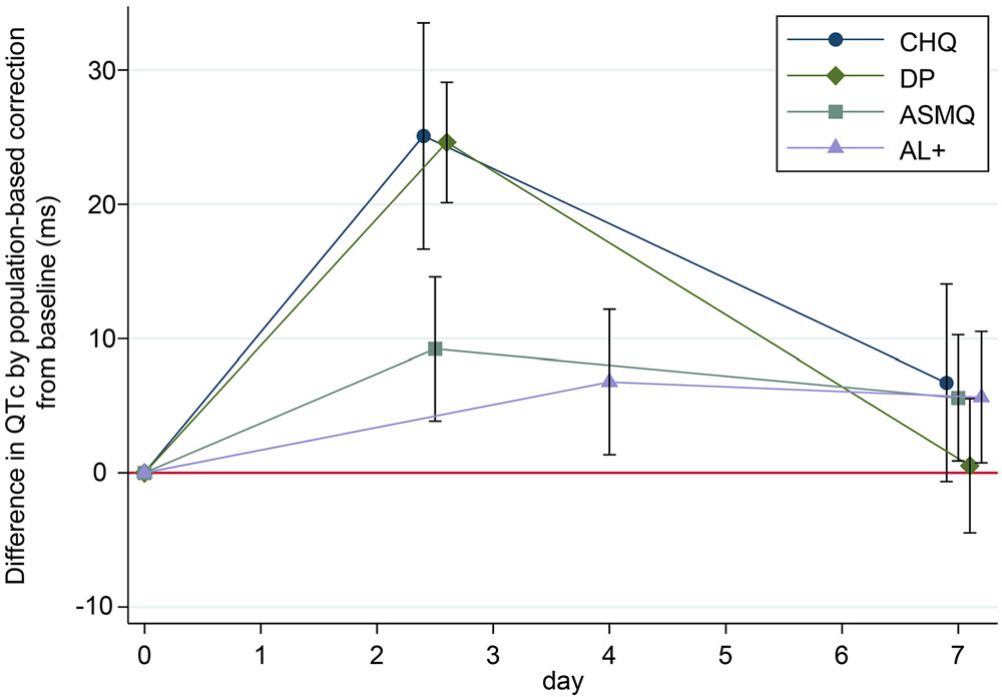
Difference in adjusted QTc by population-based correction method from baseline over time by treatment. 95% confidence intervals are shown as whiskers. AL+, extended artemether-lumefantrine; ASMQ, artesunate-mefloquine; CHQ, chloroquine; DP, dihydroartemisinin-piperaquine.

On day 7, there was no difference among the treatment groups (*P* = 0.17). QTcP on day 7 was longer than day 0 in AL+ and ASMQ, but there was no difference from day 0 in DP and CHQ (Fig. 1); the adjusted mean difference from day 0 was 5.64 ms (95% CI, 0.76 to 10.52) in AL+, 5.59 ms (95% CI, 0.90 to 10.27) in ASMQ, 0.53 ms (95% CI, −4.47 to 5.53) in DP, and 6.71 ms (95% CI, −0.64 to 14.05) in CHQ.

Variables associated with longer QTcP were age (adjusted mean difference, 0.53 ms/year; 95% CI, 0.24 to 0.81; *P* < 0.001), asexual parasite density (0.11 ms/1,000/μl; 95% CI, 0.02 to 0.19; *P* = 0.02), presence of gametocytemia (adjusted mean difference between those who had gametocytemia and those who did not, 13.53 ms; 95% CI, 3.17 to 23.90; *P* = 0.01), and diarrhea (23.16 ms; 95% CI, 8.18 to 38.14; *P* = 0.002). Increasing EGA was associated with shorter QTcP (−0.40 ms/week; 95% CI, −0.62 to −0.18; *P* < 0.001). As the time of day when ECG was assessed was a confounder for treatment effect, it was included in the final model. Although not statistically significant, QTcP was longer when the ECG was assessed between midnight and 06:00, consistent with diurnal change (Table 3).

### Sensitivity analyses.

In a sensitivity analysis excluding women who were given concomitant medications that could potentially influence QT interval (*n* = 14) or who received blood transfusions (*n* = 1), the conclusions were unchanged (Fig. S3).

To avoid overadjustment by assuming the same QTcP on day 0, a sensitivity analysis was conducted using a model that allowed a difference in the baseline QTcP on day 0 among different treatment groups. In this sensitivity analysis, the point estimates were very similar to the primary model (Fig. S3). Even in the model which allowed differences in the QTcP on day 0, there was no significant difference in the QTcP on day 0 among the treatment groups with or without adjusting for other covariates, which justifies our assumption of no difference in the primary model.

### JT interval.

The prolongation of QT interval on day peak from the baseline was mostly due to the prolongation of JT interval regardless of the correction methods (Fig. S4). No patients had bundle branch block (an abnormally wide QRS interval of >120 ms). The effect of different antimalarials on the JTc interval was similar to that on QTcP (Fig. S3). In the multivariable analysis, JTc on day peak was longer in DP (adjusted mean difference, 14.47 ms; 95% CI, 7.83 to 21.12; *P* < 0.001) and CHQ (15.24 ms; 95% CI, 5.14 to 25.33; *P* = 0.003) than AL+, but not different in ASMQ (−0.53 ms; 95% CI, −7.58 to 6.53; *P* = 0.88).

## DISCUSSION

This study in pregnant women with acute uncomplicated malaria shows that DP and CHQ prolong the electrocardiographic QT interval by a similar magnitude, consistent with observations in healthy Thai male and female (nonpregnant) adult volunteers (29, 30). DP and CHQ prolongation mainly reflect delayed ventricular repolarization as it resulted from prolongation of JT interval rather than the widening of QRS. This effect was larger than for AL+- or ASMQ-treated women.

We are not aware of any prior studies reporting ECG intervals for pregnant women receiving CHQ. No clinically relevant abnormalities have been reported in ECG studies of antimalarials in pregnant women on prevention, including mefloquine (*n* = 80) (14, 15) and DP (*n* = 492) (16–22), or treatment, including quinine (*n* = 152) (23), AL (*n* = 203) (23–25), artesunate-amodiaquine (*n* = 83) (26), and artesunate-atovaquone-proguanil (*n* = 22) (27). However, piperaquine, a bisquinoline, used in the form of DP and highly effective except in the Eastern Greater Mekong subregion, has been a focus of interest due to potential cardiotoxicity. This arose from one possibly drug-related sudden death after DP (nonpregnant) among several hundred thousand exposed individuals, a death rate that was not higher than the background population risk (31).

Malaria infection has been proposed as a cause of QTc shortening (1) due to increased sympathetic tone, and this can be independent of heart rate (32). Higher body temperature was also shown to be associated with a shorter QT interval (−2.80 ms/1°C increase; 95% credible interval, −3.17 to −2.42 ms) in malaria patients before treatment in a recent meta-analysis (7). The results presented here were generally similar in direction and magnitude with previously reported effects in nonpregnant females. The QTcP on day 0 was slightly shorter than that on day 7 for all treatment groups, and QTcP of pregnant women with higher body temperature was shorter (−1.47 ms/1°C increase; 95% CI, −4.07 to 1.13), though some of these trends did not reach statistical significance. In contrast, QTc lengthening has also been associated with intensity of malaria infection: higher parasitemia has been reported to be associated with a longer QTcB in children in one previous study (33). In the data presented here, there was a strong association between both an increasing asexual parasite density and presence of gametocytes and prolongation of the QTcP. Differences in the effect of infection on QTc by species has been reported (7) but was not found in this study.

Additional variables associated with QTc that were consistent with previous studies included prolongation of QTc with advancing age and diarrhea (34, 35) and diurnal change in QTc (36–38). Higher gestational age was associated with a shorter QTc. Similarly, one previous DP preventative treatment study (largely asymptomatic women) reported that QTc prolongation decreased in later pregnancy (21). This finding may be due to the level of progesterone (or the ratio of progesterone to estradiol), which increases toward the end of pregnancy (13, 39), or possibly the lower blood concentration (i.e., higher volume of distribution) of the partner drug later in the pregnancy (40).

QTcF was associated with heart rate in our study population. QTcB is known to overadjust QT in people with higher heart rate, which was observed in our cohort. As patients with malaria are more likely to have increased heart rate due to fever, QTcP should be a better measurement to compare drug effects in individuals where heart rate is expected to change over the treatment course.

There are some limitations to this study. Randomization was only done for AL+, ASMQ, and DP. CHQ was used for P. vivax recurrence, so some baseline characteristics, such as parasitemia load and symptoms, were not comparable with the other three drugs where allocation was randomized. The sensitivity analysis allowing difference on day 0, however, reached the same conclusion. Importantly, there was no difference in baseline QTc interval among these four drugs. Therefore, our conclusion on the drug effect, which was measured as the difference in QTcP from baseline, will not change.

In conclusion, our results indicate that the impact of DP on QTc prolongation in pregnant women with malaria was similar to that of CHQ. Although both DP and CHQ were associated with a similarly longer QTc interval on day peak than AL+ or ASMQ, the recorded longest QTc interval did not exceed the known threshold for increased risk for fatal arrhythmia. The drug effect on QTc did not remain on day 7. This study thus demonstrated a safe cardiotoxic profile of the currently used antimalarials (namely, AL+, ASMQ, DP, and CHQ) for treating uncomplicated malaria in pregnancy.

## MATERIALS AND METHODS

### Study site and eligibility criteria.

This study was a part of a randomized controlled trial (RCT) in pregnancy conducted from 2010 to 2016 on the Thailand-Myanmar border, details of which are described elsewhere (ClinicalTrials.gov identifier NCT01054248). Briefly, pregnant women were screened for malaria parasites by blood smear at the first antenatal consultation and then every 2 weeks, and women with positive parasitemia were assessed for eligibility. Inclusion criteria were age 18 to 45 years with a viable fetus confirmed by ultrasound, microscopically confirmed uncomplicated malaria of any species with a parasitemia of ≥5/500 white blood cells, and no signs of labor. Patients with severe malaria, hyperparasitemia (≥4%), severe anemia (hematocrit < 20%), or known history of chronic diseases were excluded. After giving informed consent in their own language, pregnant women were randomized to take either AL+, ASMQ, or DP. The treatment allocation was concealed using sealed opaque envelopes. A computer-generated randomization of 1:1:1 in blocks of 15 was used. This RCT was open-label: only the readers of the ECG were blinded for the treatment allocation.

Drugs were given under full supervision for all doses. AL+ was given at a higher dose and for a longer period than the current standard: five tablets of Coartem (20/120 mg artemether/lumefantrine) were given twice a day for 4 days regardless of body weight, with 100 ml of chocolate milk each time. ASMQ was given once daily for 3 days as either a loose combination of artesunate (50 mg/tablet) plus mefloquine (250 mg/tablet) or a fixed-dose combination (artesunate/mefloquine, 100/220 mg) depending on availability. For the loose combination, the dose was rounded to the nearest quarter of a tablet for both drugs based on body weight (4 mg/kg artesunate and 8 mg/kg mefloquine). For the fixed-dose combination, two tablets were given each day to all women (with body weight >29 kg). DP was given based on body weight (2.4 mg/kg dihydroartemisinin and 20 mg/kg piperaquine) once daily for 3 days. Standard fixed-dose tablets containing 40/320 mg of dihydroartemisinin/piperaquine were used, and the dose was rounded up to the nearest half of a tablet. Recurrence of non-P. falciparum parasitemia during follow-up was treated with chloroquine (10 mg/kg on day 0 and 1 and 5 mg/kg on day 2) with the dose rounded to the nearest quarter of a tablet (one tablet contained 250 mg chloroquine phosphate).

After treatment, women were followed up weekly for clinical, obstetric, and parasitological assessment until delivery or for 63 days, whichever was later.

### ECG assessment.

A standard 12-lead ECG (Nihon Kohden ECG-1250K; Tokyo, Japan) was measured at baseline before drug administration (day 0), at 4 to 6 h following the last dose (defined as day peak), and on day 7 or after (day 7). ECG was assessed in a supine position after a short rest in the same position. ECGs were only assessed in around half the patients (259/511) enrolled in this study, depending on the availability of ECG machines at the study sites.

One assessor, who was blinded to the treatment allocation, read all ECG records manually. Another assessor independently read the ECGs of the first 180 patients. The end of the T wave was defined as the point of intersection between the isoelectric line and the tangent line to the steepest downslope of the T wave. If the U wave was fused with the T wave and its height was more than half of that of the T wave, the U wave was included for measurement of the QT (41). In total, six QRS complexes were measured using at least four different leads, and the median of these six complexes was taken (42). The minimum precision of reading was 0.5 mm, which corresponded to 10 ms at a speed of 50 mm/s. The heart rate given by the ECG machine was used to calculate the average RR interval (42). QRS interval was measured automatically by the machine or manually if the automatic reading was not provided. All concomitant medications used during the ECG assessment period were recorded.

### Statistical analysis.

Proportions of the study population with QT prolongation on day 0, day peak, and day 7 were summarized by treatment, using QTu, QTcF, QTcB, and QTcP (43). QT prolongation was defined either by the absolute value of >480 ms or >500 ms, or by the absolute increase of >30 ms or >60 ms from baseline (5, 44).

To compare the QT intervals with different heart rates, a correction method specifically for this study population (QTcP) was derived from a linear regression of log(QT) and log(RR) using the data on day 0 (45). Either QTcF, QTcB, or QTcP that was the least related to heart rate was used for further analyses.

Linear mixed-effects models were used for the analyses. For interpretation, coefficients of linear regression were expressed as the predicted mean difference. Repeated measures analyses were conducted for QTc at different time points. The model assumed that there was no difference in the baseline QTc (46). Variable selection was done by backward elimination (47) using a *P* value of 0.05 by the Wald test as the cutoff. Variables that changed the predicted mean difference of treatment by more than 10% were kept in the multivariable model regardless of the *P* value if clinically considered a confounder (48). Symptoms that are known to be (or are potentially) associated with QT prolongation were assessed as covariates. Two-level random intercepts were used for within-person correlation (46).

The following three types of sensitivity analyses were conducted, including all variables in the final multivariable model: a model excluding episodes with any concomitant drugs known to be associated with QT prolongation (https://www.crediblemeds.org/) used, a model allowing the difference in QTc on day 0 (46), and a model using the corrected JT interval, which is defined as QTc − QRS (49), as the outcome.

Stata/MP 16.1 (Stata Corp., TX, USA) was used for the statistical analyses.

### Ethics.

After the discussion with the Tak Province Border Community Ethics Advisory Board (T-CAB) (50), this study was approved by The Ethical Committee of the Faculty of Tropical Medicine, Mahidol University in Bangkok (TMEC 09-050) and the Oxford Tropical Research Ethics Committee (OXTREC 45-09). This study is registered in clinicaltrials.gov (NCT01054248).

### Data availability.

Data are available from MORU Tropical Health Network (https://www.tropmedres.ac/units/moru-bangkok/bioethics-engagement/data-sharing).

## Supplementary Material

Supplemental file 1
