# Supplementary material for: Randomized Controlled Trial of the Electrocardiographic Effects of Four Antimalarials for Pregnant Women with Uncomplicated Malaria on the Thailand-Myanmar Border
Source: Antimicrob Agents Chemother. 2021 Mar 18;65(4):e02473-20. doi: 10.1128/AAC.02473-20 (PMC8097415; doi:10.1128/AAC.02473-20)
Supplement: Supplemental file 1 [file AAC.02473-20-s0002.pdf]

Supplementary Table 1. Baseline characteristics comparing those who were assessed by ECG and those who were not

Characteristic	ECG assessed (ACTs)		ECG not-assessed (ACTs)		ECG assessed (CHQ)		ECG not-assessed (CHQ)	
	N	Mean (SD) / % (N)	N	Mean (SD) / % (N)	N	Mean (SD) / % (N)	N	Mean (SD) / % (N)
Age (years)	256	25.5 (6.9)	255	26.2 (8.1)	18	25.1 (7.6)	31	25.5 (6.9)
Gravidity	256	2.9 (2.0)	255	3.1 (2.5)	18	3.1 (2.0)	31	2.7 (1.9)
Height (cm)	256	151.1 (5.6)	255	150.6 (8.2)	18	150.3 (5.4)	31	149.0 (19.0)
Smoking	256	20.3% (52)	255	19.2% (49)	18	22.2% (4)	31	16.1% (5)
Gestational age (weeks)	256	25.6 (8.4)	255	25.9 (7.9)	18	28.7 (6.1)	31	24.5 (10.4)
Weight (kg)	256	52.0 (7.9)	255	51.5 (7.3)	17	55.1 (7.4)	31	50.7 (8.7)
Fever (>37.5°C)	256	31.6% (81)	255	23.1% (59)	18	16.7% (3)	31	22.6% (7)
Pulse rate (/beats per min)	254	91.3 (12.3)	246	89.8 (10.8)	18	84.3 (9.2)	31	89.9 (15.2)
Haematocrit (%)	256	32.5 (3.8)	255	31.8 (4.2)	18	31.8 (2.6)	31	33.1 (2.9)
Malaria species	256		255		18		31	
falciparum monoinfection		30.1% (77)		25.5% (65)		0.0% (0)		0.0% (0)
vivax monoinfection		67.6% (173)		70.6% (180)		100.0% (18)		100.0% (31)
falciparum + vivax		2.0% (5)		3.9% (10)		0.0% (0)		0.0% (0)
malariae monoinfection		0.4% (1)		0.2% (0)		0.0% (0)		0.0% (0)
Asexual parasite load	256	10962.0 (22157.2)	255	11257.0 (25512.7)	18	2696.0 (6895.7)	31	6129.9 (17947.1)
Presence of gametocyte	256	46.9% (120)	255	53.7% (137)	18	72.2% (13)	31	74.2% (23)
Anorexia	256	38.7% (99)	254	49.2% (125)	18	22.2% (4)	30	13.3% (4)
Nausea	256	34.4% (88)	255	31.4% (80)	18	0.0% (0)	30	10.0% (3)
Vomiting	256	17.2% (44)	255	19.2% (49)	18	0.0% (0)	30	6.7% (2)
Dizziness	256	64.5% (165)	255	59.6% (152)	18	50.0% (9)	30	43.3% (13)
Diarrhoea	256	2.3% (6)	255	1.6% (4)	18	0.0% (0)	30	0.0% (0)
Palpitation	256	34.0% (87)	255	30.2% (77)	18	0.0% (0)	30	13.3% (4)
Fatigue	256	53.9% (138)	255	42.0% (107)	18	50.0% (9)	30	20.0% (6)
Treatment	256		255		-		-	
AL+		33.6% (86)		32.9% (84)				
ASMQ		34.4% (88)		32.9% (84)				
DP		32.0% (82)		34.1% (87)				

ACTs: artemisinin-based combination therapies, AL+: extended artemether-lumefantrine, ASMQ: artesunate-mefloquine, CHQ; chloroquine, DP: dihydroartemisinin-piperaquine, ECG: electrocardiogram, SD: standard deviation.

Supplementary Table 2. QT interval on day 0, day peak and day 7 by different correction methods for each treatment

Measurement	All		AL+		ASMQ		DP		CHQ	
	Total	Mean (SD)<br>Range	Total	Mean (SD)<br>Range	Total	Mean (SD)<br>Range	Total	Mean (SD)<br>Range	Total	Mean (SD)<br>Range
**Day 0**										
Heart rate (/min)	268	93.4 (16.2)<br>58–159	83	96.2 (17.3)<br>63–159	78	95.6 (16.2)<br>61–137	86	90.3 (15.2)<br>58–132	19	86.8 (12.6)<br>61–112
QT uncorrected	268	334.3 (27.4)<br>250.0–415.0	83	330.4 (30.5)<br>250.0–390.0	78	332.6 (26.4)<br>275.0–415.0	86	337.6 (26.0)<br>280.0–395.0	19	342.4 (21.6)<br>300.0–380.0
QTc Fridericia	268	384.8 (20.4)<br>322.7–436.3	83	383.8 (21.7)<br>322.7–428.3	78	385.9 (21.2)<br>335.2–431.9	86	384.5 (19.3)<br>338.2–436.3	19	385.6 (17.5)<br>348.5–423.4
QTc Bazett	268	413.4 (23.4)<br>355.9–486.7	83	414.1 (22.0)<br>355.9–467.1	78	416.2 (25.9)<br>360.1–486.7	86	410.8 (22.9)<br>359.1–478.5	19	409.6 (21.6)<br>360.9–446.9
QTcP	268	392.8 (20.6)<br>334.7–447.2	83	392.2 (21.2)<br>334.7–439.1	78	394.3 (22.0)<br>343.9–446.9	86	391.8 (19.7)<br>344.1–447.2	19	392.3 (18.2)<br>352.0–430.0
**Day peak**										
Heart rate (/min)	254	78.6 (11.8)<br>48–120	82	78.7 (13.0)<br>48–111	77	79.9 (12.8)<br>57–120	79	77.7 (10.1)<br>59–107	16	76.8 (8.3)<br>59–90
QT uncorrected	254	367.1 (28.1)<br>285.0–445.0	82	361.5 (28.9)<br>290.0–435.0	77	359.8 (27.5)<br>285.0–420.0	79	377.8 (25.5)<br>300.0–445.0	16	377.8 (21.7)<br>335.0–410.0
QTc Fridericia	254	399.6 (21.1)<br>342.6–461.9	82	393.1 (18.9)<br>352.3–438.2	77	393.6 (20.8)<br>342.6–447.0	79	410.2 (19.9)<br>363.7–461.9	16	409.0 (15.2)<br>362.5–425.3
QTc Bazett	254	417.3 (23.0)<br>360.1–493.3	82	410.5 (21.4)<br>360.6–470.6	77	412.1 (23.0)<br>360.1–471.5	79	427.7 (21.6)<br>377.6–493.3	16	425.7 (16.7)<br>377.1–444.1
QTcP	254	404.5 (21.2)<br>347.5–470.7	82	398.0 (18.9)<br>355.6–447.3	77	398.8 (21.0)<br>347.5–451.8	79	415.1 (20.0)<br>373.3–470.7	16	413.7 (15.2)<br>366.7–428.0
**Day 7**										
Heart rate (/min)	226	86.2 (12.9)<br>52–124	71	86.9 (13.3)<br>52–124	68	83.1 (12.4)<br>58–112	67	86.9 (13.0)<br>65–123	20	92.2 (9.8)<br>75–109
QT uncorrected	226	346.0 (26.7)<br>285.0–420.0	71	346.3 (28.9)<br>290.0–420.0	68	352.2 (26.9)<br>285.0–410.0	67	341.5 (26.4)<br>290.0–400.0	20	339.5 (12.0)<br>320.0–360.0
QTc Fridericia	226	388.3 (19.1)<br>335.7–429.3	71	389.3 (18.2)<br>349.6–429.3	68	390.5 (20.2)<br>335.7–428.4	67	384.3 (19.6)<br>336.9–423.7	20	391.0 (15.5)<br>350.8–416.4
QTc Bazett	226	411.8 (21.4)<br>352.2–460.6	71	413.3 (18.9)<br>380.8–460.6	68	411.6 (23.1)<br>352.2–456.0	67	408.0 (21.6)<br>357.0–456.0	20	419.9 (21.7)<br>367.3–454.1
QTcP	226	394.9 (19.2)<br>340.3–434.5	71	396.0 (17.8)<br>359.1–434.5	68	396.4 (20.5)<br>340.3–431.9	67	390.9 (19.7)<br>342.6–429.6	20	399.1 (17.0)<br>355.5–426.9

AL+: extended artemether-lumefantrine, ASMQ: artesunate-mefloquine, CHQ; chloroquine, Δ: difference, DP: dihydroartemisinin-piperaquine, QTc Fridericia: QT interval corrected by Fridericia method, QTc Bazett: QT interval corrected by Bazett method, QTcP: QT interval corrected by population-based correction ($QT/RR^{0.381}$), SD: standard deviation.

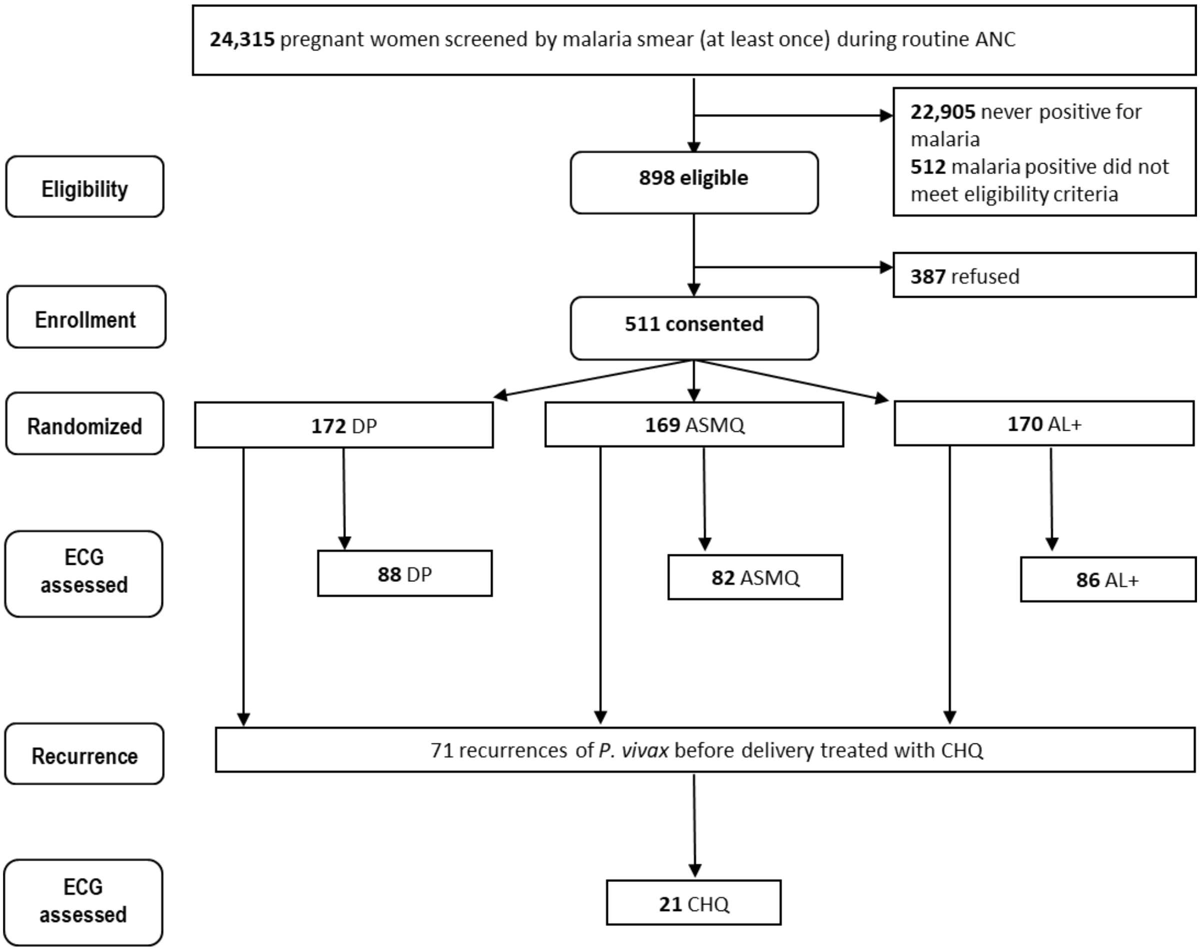

Supplementary Figure 1. Flowchart of the pregnant women enrolled in the randomised controlled study and assessed for ECG

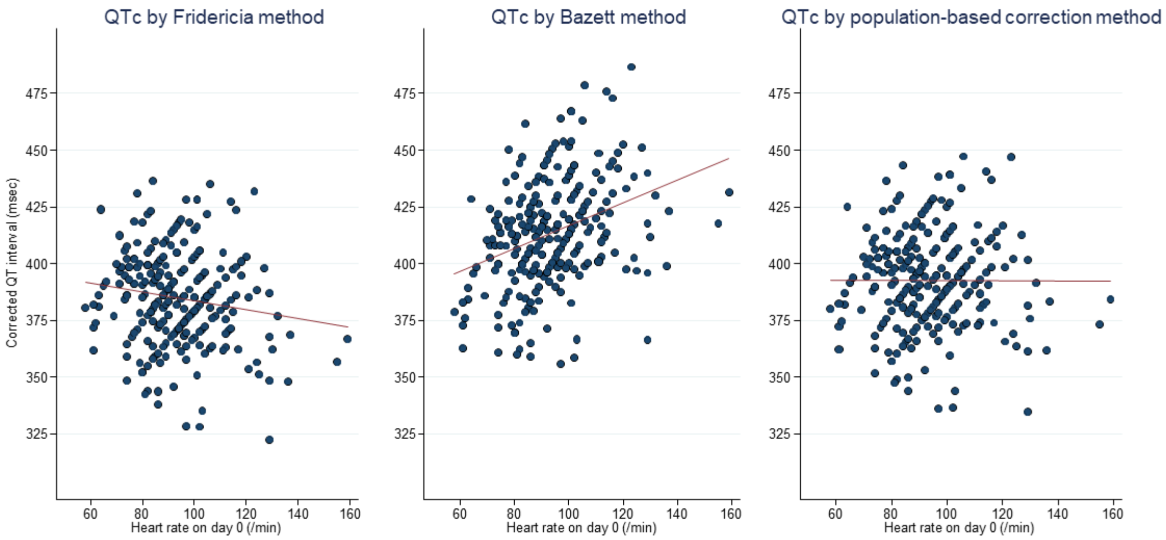

Supplementary Figure 2. Comparison between corrected QT interval (QTc) by Fridericia, Bazett and population-based correction method

Linear relationship remains after the Fridericia or Bazett correction, but not after population-based correction ($QT/RR^{0.381}$).

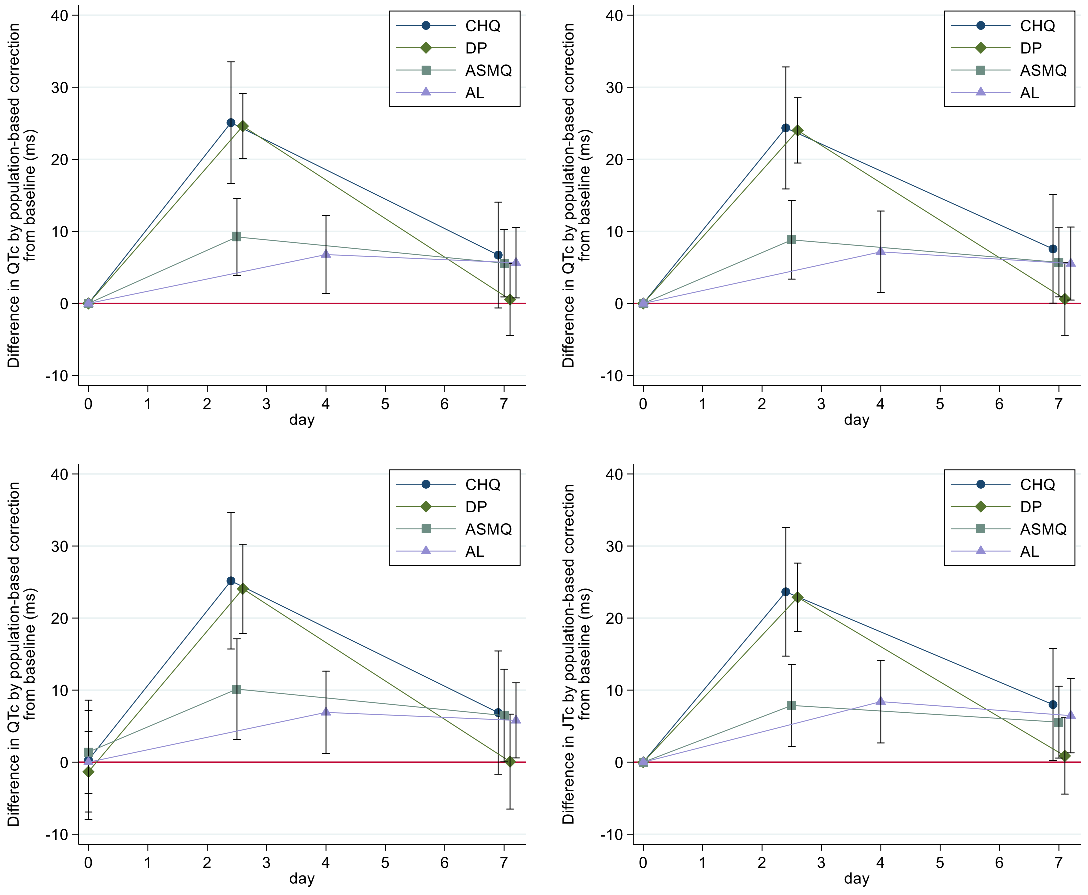

Supplementary Figure 3. Adjusted QTc by population-based correction method over time by treatment in the final model and three sensitivity analysis models

The main model adjusting for the baseline (top left, the same as Figure 2), a model excluding those who used concomitant medication (top right), a model allowing the baseline difference (bottom left), and a model with JTc interval as the outcome (bottom right).

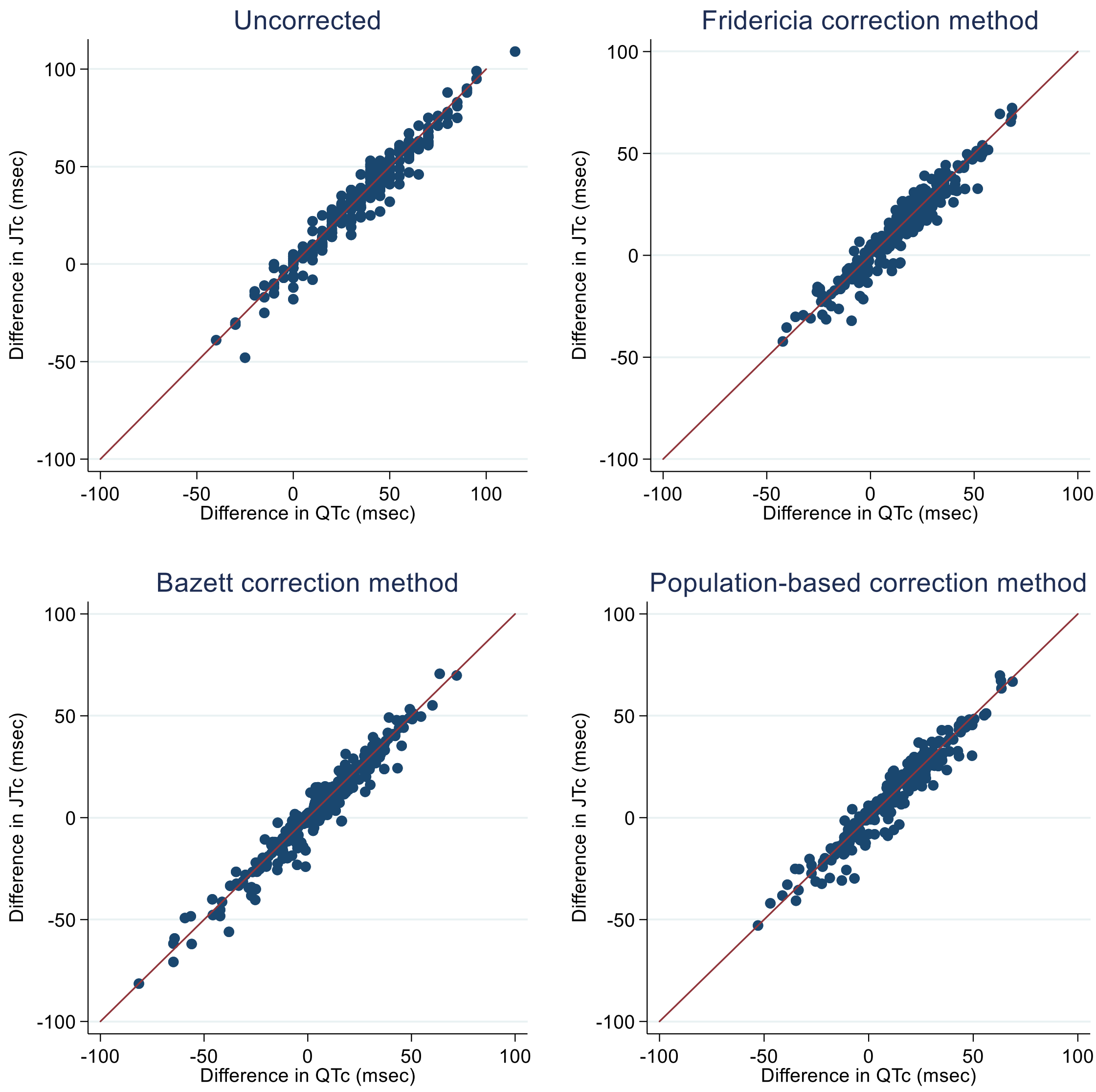

Supplementary Figure 4. Difference in QT interval and JT interval on day peak from the baseline.

QT and JT intervals are either uncorrected (top left), corrected by Fridericia method (top right), by Bazett method (bottom left), and by population-based correction method ($QT/RR^{0.381}$, bottom right). Red line shows y=x.
